# Supplementary material for: Analysis of PPARγ Signaling Activity in Psoriasis
Source: Int J Mol Sci. 2021 Aug 10;22(16):8603. doi: 10.3390/ijms22168603 (PMC8395241; doi:10.3390/ijms22168603)
Supplement: Supplementary file 1 [file ijms-22-08603-s001.zip › Supplemental materials_Analysis of PPARg signaling activity in psoriasis/Pathway models/Models images and html files/PPARG inhibits phenotypes assotiated with psoriasis/136928.html]

osteoarthritis


# Disease osteoarthritis

|  |  |
| --- | --- |
| URN | urn:agi-meshdis:Osteoarthritis |
| Total Entities | 7 |
| Connectivity | 5002 |
| Name | osteoarthritis |

---

|  |  |
| --- | --- |
| ChildConcepts | spondylosis |
|  | hand osteoarthritis |
|  | experimental osteoarthritis |
|  | arthrosis |
|  | hip osteoarthritis |
|  | knee osteoarthritis |
|  | premature osteoarthritis |

---

|  |  |
| --- | --- |
| Pathway | Proteins Involved in Osteoarthritis |
|  | Genes with Mutations Associated with Osteoarthritis |
|  | Proteins with Altered Expression in Osteoarthritis |
|  | PPARG inhibits phenotypes assotiated with psoriasis |
|  | Neighbors of osteoarthritis |
|  | Overview. Diseases of the musculoskeletal system (chapter 12) |

---

|  |  |
| --- | --- |
| MedScan ID | 9016114 |

---

|  |  |
| --- | --- |
| Alias | osteo-arthrosis |
|  | osteo-arthritic |
|  | Degenerative Arthritis |
|  | Osteo-athritis |
|  | Osteoarthritides |
|  | Osteoarthrosis |
|  | Arthritis, Degenerative |
|  | Arthropathy, Degenerative |
|  | Arthropathies, Degenerative |
|  | Degenerative Arthropathy |
|  | Osteoarthroses |
|  | Osteoarthritic |
|  | primary osteoarthritis |
|  | Osteo-athrites |
|  | Osteoathrites |
|  | Degenerative Arthritides |
|  | degenerative arthropathies |
|  | Osteoathritis |
|  | Arthritis, Hypertrophic |
|  | Hypertrophic Arthritis |
|  | Osteoarthritis |
|  | Degenerative Joint Diseases |
|  | Degenerative Polyarthritis |
|  | Osteo-Arthritis |
|  | Degenerative Joint Disease |

---

|  |  |
| --- | --- |
| MeSH ID | D010003 |
|  | M0015509 |

---

|  |  |
| --- | --- |
| MeSH Heading | Osteoarthritis |

---
